# Applying the WHO-INTEGRATE evidence-to-decision framework in the development of WHO guidelines on parenting interventions: step-by-step process and lessons learnt

**DOI:** 10.1186/s12961-024-01165-z

**Published:** 2024-07-05

**Authors:** Ani Movsisyan, Sophia Backhaus, Alexander Butchart, Frances Gardner, Brigitte Strahwald, Eva Rehfuess

**Affiliations:** 1 Chair of Public Health and Health Services Research, Faculty of Medicine, Institute for Medical Information Processing, Biometry and Epidemiology (IBE), LMU Munich, Elisabeth-Winterhalter-Weg 6, 81377 Munich, Germany; 2 Pettenkofer School of Public Health, Elisabeth-Winterhalter-Weg 6, 81377 Munich, Germany; 3 Centre for Evidence-Based Intervention, Department of Social Policy and Intervention, University of Oxford, 32 Wellington Sq., Oxford, OX1 2ER United Kingdom; 4 Violence Prevention Unit, Social Determinants of Health Department, Healthier Populations Division, World Health Organization, Geneva, Switzerland

**Keywords:** Evidence-to-decision framework, Guideline development, Public health, Health policy, Complex systems thinking, Parenting interventions.

## Abstract

**Background:**

Development of guidelines for public health, health system, and health policy interventions demands complex systems thinking to understand direct and indirect effects of interventions within dynamic systems. The WHO-INTEGRATE framework, an evidence-to-decision framework rooted in the norms and values of the World Health Organization (WHO), provides a structured method to assess complexities in guidelines systematically, such as the balance of an intervention’s health benefits and harms and their human rights and socio-cultural acceptability. This paper provides a worked example of the application of the WHO-INTEGRATE framework in developing the WHO guidelines on parenting interventions to prevent child maltreatment, and shares reflective insights regarding the value added, challenges encountered, and lessons learnt.

**Methods:**

The methodological approach comprised describing the intended step-by-step application of the WHO-INTEGRATE framework and gaining reflective insights from introspective sessions within the core team guiding the development of the WHO guidelines on parenting interventions and a methodological workshop.

**Results:**

The WHO-INTEGRATE framework was used throughout the guideline development process. It facilitated reflective deliberation across a broad range of decision criteria and system-level aspects in the following steps: (1) scoping the guideline and defining stakeholder engagement, (2) prioritising WHO-INTEGRATE sub-criteria and guideline outcomes, (3) using research evidence to inform WHO-INTEGRATE criteria, and (4) developing and presenting recommendations informed by WHO-INTEGRATE criteria. Despite the value added, challenges, such as substantial time investment required, broad scope of prioritised sub-criteria, integration across diverse criteria, and sources of evidence and translation of insights into concise formats, were encountered.

**Conclusions:**

Application of the WHO-INTEGRATE framework was crucial in the integration of effectiveness evidence with insights into implementation and broader implications of parenting interventions, extending beyond health benefits and harms considerations and fostering a whole-of-society-perspective. The evidence reviews for prioritised WHO-INTEGRATE sub-criteria were instrumental in guiding guideline development group discussions, informing recommendations and clarifying uncertainties. This experience offers important lessons for future guideline panels and guideline methodologists using the WHO-INTEGRATE framework.

**Supplementary Information:**

The online version contains supplementary material available at 10.1186/s12961-024-01165-z.

## Key questions

**What is already known on this topic**

- Evidence-to-decision (EtD) frameworks are an important tool in guideline development, bridging systematic evidence evaluation and decision-making, and emphasising intervention effectiveness and adverse effects, as well as other criteria like resource use, feasibility, and acceptability in informing practice recommendations.

**What this study adds**

- This study demonstrates the practical application of the WHO-INTEGRATE framework in developing multi-sectoral WHO guidelines on parenting interventions, showing how complex systems thinking is put into practice in a comprehensive manner and substantiated through both evidence synthesis and careful deliberation by the guideline development group.
- It reveals the added value and challenges of employing the WHO-INTEGRATE framework and offers specific recommendations for guideline development groups/guideline panels and guideline methodologists in future public health, health system, and health policy guideline development.

**How this study might affect research, practice or policy**

- This study underscores the importance of incorporating complex systems thinking and a broader set of considerations in guideline development, potentially shifting research, practice, and policy towards more holistic and context-sensitive approaches.

## Background

Guidelines represent an important tool to support evidence-based decision-making, and are employed by many national technical agencies around the world, including the World Health Organization (WHO), to develop practice recommendations and enable their implementation. In this context, evidence-to-decision (EtD) frameworks provide a structured approach for bringing scientific evidence into policy and practice recommendations [1]. These frameworks bridge the systematic evaluation of evidence and decision-making, ensuring that guidelines are grounded in the best available evidence on intervention effectiveness and adverse effects, and consider other factors, such as resource implications, feasibility, acceptability, and equity [2]. By facilitating systematic deliberation of an agreed set of decision criteria, EtD frameworks enhance the transparency, applicability, and legitimacy of guidelines [3].

Developing guidelines for public health, health system, and health policy interventions presents unique challenges, necessitating a shift towards complex systems thinking [4]. Unlike clinical guidelines that tend to address the diagnosis and treatment of individual patients, public health guidelines grapple with multifaceted problems embedded in social, economic, and environmental systems [2, 4]. Relevant interventions frequently require coordinated actions across multiple sectors and levels of governance, making the traditional linear approach to clinical guideline development insufficient. A complex systems perspective enables guideline developers to understand and anticipate the many indirect effects and interactions that may arise within the dynamic systems in which public health, health system and health policy interventions are implemented [4].

The WHO-INTEGRATE framework provides a new tool by which such complexities can be systematically assessed, and their policy and practice implications unpacked. Rooted in the norms and values of the WHO, the framework builds upon existing EtD methodologies [1, 3] and integrates a broader set of decision criteria particularly relevant to public health, health system and health policy interventions [2, 5]. Specifically, the framework comprises six substantive criteria—balance of health benefits and harms, human rights and sociocultural acceptability, health equity, equality and non-discrimination, societal implications, financial and economic considerations, and feasibility and health system considerations—and the meta-criterion quality of evidence, which relates to each of the substantive criteria (see Table 1). It is designed to enable a rigorous, reflective, and contextualised deliberation from the outset of guideline development, and is particularly well suited for guidelines focusing on population- and system-level interventions [2, 4].

**Table 1:** WHO-INTEGRATE criteria and sub-criteria: adaptation in WHO parenting guidelines

Criterion	Sub-criteria	Application	
		Yes	No
Balance of health benefits and harms	Efficacy or effectiveness on the health of individuals	–	
	Effectiveness or impact on the health of populations	–	
	Beneficiaries’ values in relation to health outcomes*		–
	Safety-risk profile	–	
	Broader positive or negative impacts	–	
Human rights and socio-cultural acceptability	Accordance with universal human rights	–	
	Socio-cultural acceptability to beneficiaries	–	
	Socio-cultural acceptability to implementers	–	
	Socio-cultural acceptability to the public	–	
	Impact on autonomy	–	
	Intrusiveness	–	
Health equity, equality, and non-discrimination	Impact on health equity/equality	–	
	Distribution of benefits and harms	–	
	Affordability	–	
	Accessibility	–	
	Severity and/or rarity of the condition		–
	Lack of suitable alternative		–
Societal implications	Social impact	–	
	Environmental impact		
Financial and economic considerations	Financial impact	–	–
	Impact on economy	–	
	Ratio of costs and benefits	–	
Feasibility and health system considerations	Legislation	–	
	Leadership and governance	–	
	Interaction with and impact on health system	–	
	Need for, usage of and impact on health workforce and human resources	–	
	Need for, usage of and impact on infrastructure	–	

*This sub-criterion was integrated with “socio-cultural acceptability to beneficiaries”

In 2022, WHO published guidelines providing evidence-based recommendations on parenting interventions to prevent child maltreatment and enhance parent–child relationships (hereafter referred to as “WHO parenting guidelines”) [6]. These describe key components of effective parenting interventions, emphasising their role in enhancing positive parenting behaviours and reducing child maltreatment, harsh parenting, and behavioural and mental health issues in children, and their positive impact on parental mental health and stress reduction. Applicable globally, the guidelines are intended for a diverse audience, including policymakers, development agencies, implementing partners, health and social workers, and non-governmental organisations across low- and middle-income countries (LMIC), as well as high-income countries (HIC). Given the complex and multi-sectoral nature of parenting interventions (e.g. health, education, social services), the WHO-INTEGRATE framework was used throughout the guideline development process. Parenting interventions, contributing to the wellbeing of children and societies at large, were evaluated against all six WHO-INTEGRATE criteria, and the recommendations were rooted in several comprehensive evidence reviews keyed to these criteria [7, 8].

## Objectives

In this paper, we describe the process of applying the WHO-INTEGRATE framework in developing the WHO parenting guidelines. Our objectives are to (i) provide a worked example of the steps involved in applying the WHO INTEGRATE framework in a guideline development process, and (ii) share reflective insights regarding the value added, challenges encountered, and lessons learnt. This is intended to aid guideline development groups (GDGs)/guideline panels and guideline methodologists in future use of the framework.

## Methods

To achieve these objectives, we followed a methodological approach that comprised (i) describing the intended steps in the application of the WHO-INTEGRATE framework in guideline development, and (ii) gaining reflective insights through (a) introspective sessions within the WHO parenting guideline core team on the actual application of the framework, and (b) preparing and conducting a methodological workshop on the WHO-INTEGRATE framework.

### Intended steps in the application of the WHO-INTEGRATE framework in guideline development

Below we describe overarching steps in guideline development, and the key elements of the ‘intended’ application of the WHO-INTEGRATE framework in these steps [2, 4, 9]; Table 2 provides an overview of the role the WHO-INTEGRATE framework plays in each of these steps. In the Results, we detail our ‘actual’ application of the WHO-INTEGRATE framework within the WHO parenting guidelines, highlighting challenges encountered and lessons learnt.

**Table 2:** WHO-INTEGRATE framework: generic step-by-step application

Step 1	Scoping the guideline and defining stakeholder engagement
	• Definition of guideline questions
	• Choice of guideline perspective
	• Choice of EtD framework
	• Evidence mapping
	• Development of logic model(s)
	• Preliminary outcome listing
	• GDG/guideline panel deliberation
Step 2	Prioritising WHO-INTEGRATE sub-criteria and prioritising guideline outcomes
	• Discussion and prioritisation of WHO-INTEGRATE criteria and sub-criteria
	• Outcome ranking
Step 3	Using research evidence to inform WHO-INTEGRATE criteria
	• Translation of prioritised sub-criteria into research questions
	• Deliberations on evidence synthesis and/or rapid approaches
	• Commissioning and conduct of evidence syntheses
Step 4	Developing and presenting recommendations informed by WHO-INTEGRATE criteria
	• Preparation of preliminary EtD tables
	• Drafting recommendations
	• GDG/guideline panel deliberation
	• Finalisation of the EtD tables and recommendations

*EtD* evidence-to-decision, *GDG* guideline development group

### Step 1: Scoping the guideline and defining stakeholder engagement

Guideline scoping determines the guideline’s direction and focus. The ***objectives*** of this step include: (i) identifying guideline questions, (ii) deciding on the guideline perspective and choosing an appropriate EtD framework, and (iii) laying the groundwork for the entire guideline development process [8]. Various approaches can assist in the ***process*** of guideline scoping. These include evidence mapping, logic modelling [10], GDG/guideline panel reflections on the relevance of WHO-INTEGRATE criteria, and stakeholder consultations [4]. ***Outputs*** include (i) a clear guideline perspective (e.g. whether the guideline adopts a complex systems perspective), (ii) guideline questions about intervention effectiveness (formulated according to the Population, Intervention, Comparator, Outcomes [PICO] format) and broader questions informed by WHO-INTEGRATE criteria, and (iii) a preliminary list of relevant outcomes.

### Step 2: Prioritising WHO-INTEGRATE sub-criteria and prioritising outcomes

All six substantive criteria of the WHO-INTEGRATE framework, and the meta-criterion quality of evidence, are important and should be considered in all guidelines. However, the sub-criteria (see Table 1), which are designed to facilitate implementation of each criterion, should be used selectively, as it is usually neither relevant nor feasible to consider all of them. The guideline perspective will inform which outcomes (e.g. intermediate outcomes on the pathway to desired health outcomes) are considered relevant. Prioritised outcomes must be considered in systematic reviews of the effectiveness and adverse effects of interventions. Some – but not all – WHO-INTEGRATE criteria can be operationalised as outcomes. The ***objectives*** of this step include (i) selecting the most relevant WHO-INTEGRATE sub-criteria and (ii) identifying the most relevant outcomes. Concerning the first objective, this ***process*** may involve informal discussions within the GDG/guideline panel or a more formal procedure (e.g. a ranking method). With regards to the second objective, the importance of outcomes is formally rated [11]. ***Outputs*** include (i) a list of prioritised WHO-INTEGRATE sub-criteria and (ii) a maximum of seven important or critical outcomes.

### Step 3: Using research evidence to inform WHO-INTEGRATE criteria

Ideally, the WHO-INTEGRATE framework is populated with research evidence for all prioritised sub-criteria; however, this is often not feasible. The research approach needs to be fit-for-purpose (e.g., considering the human rights criterion may require a legal assessment; assessing social acceptability may need qualitative data synthesis). Therefore, the ***objectives*** of this step include (i) formulating questions derived from the prioritised sub-criteria, (ii) determining appropriate evidence synthesis (e.g. qualitative systematic review) or more feasible rapid approaches (e.g. survey) to address them, and (iii) conducting the synthesis, appraisal, and grading of evidence. The ***process*** for the first two objectives may involve informal discussions within the GDG/guideline panel, such as brainstorming sessions to weigh different options along with their advantages and disadvantages, or a more formalised voting process. ***Outputs*** include evidence products that align with the prioritised sub-criteria.

### Step 4: Developing and presenting recommendations informed by WHO-INTEGRATE criteria

Available evidence regarding WHO-INTEGRATE criteria must be presented in a transparent and comprehensible manner to facilitate deliberations and decisions by the GDG/guideline panel. This usually entails preparing detailed EtD tables. With regards to the guideline document, an accessible summary of the rationale for the recommendations is likely more appropriate for a broad readership. The ***objectives*** of this step are (i) preparing preliminary EtD tables that display the evidence supporting each prioritised sub-criterion, (ii) formulating recommendations through GDG/guideline panel deliberation and weighing the different criteria against each other, (iii) determining the strength of the recommendations, (iv) finalising the EtD tables, and (v) presenting the rationale for judgements on criteria/sub-criteria in an accessible manner. The ***process*** to accomplish this involves in-depth engagement of the GDG/guideline panel with the EtD tables prior to and during meetings, supplemented by more structured voting procedures as needed. Additionally, iterative discussions, revisions, and the collection of feedback through post-meeting communication may be helpful. ***Outputs*** include (i) the finalised EtD tables and (ii) the definitive guideline recommendations with their supporting rationale.

### Reflective insights regarding the application of the WHO-INTEGRATE framework

#### Introspective sessions on the actual application of the WHO-INTEGRATE framework

We engaged in introspective sessions on the added value of the WHO-INTEGRATE framework and challenges with its application within the WHO parenting guideline core team, comprising the WHO secretariat (AB), the guideline methodologists (ER, AM), and the leads of evidence synthesis (FG and SB). Sessions took place as a mix of smaller meetings (ER, AM), full-group virtual meetings and learning during the writing process. These deliberations were instrumental in extracting critical lessons from our experience with the framework.

#### Methodological workshop on the WHO-INTEGRATE framework

Several co-authors (ER, BS, AM) convened a methodological workshop on the WHO-INTEGRATE framework in Geneva on 22–23 November 2022. With a methods-focused group of participants (i.e. several methodologists supporting the development of guidelines at WHO and elsewhere), the workshop objectives were to advance users’ proficiency in the application of the framework, including based on the experience with the WHO parenting guidelines, and provide a platform for dialogue on the challenges with and potential enhancements to framework application. Insights gleaned from the preparatory phase and discussions during the workshop identified the framework's benefits as well as challenges encountered in its application.

## Results

### Overview of development process of the WHO parenting guidelines

**Fig. 1:**
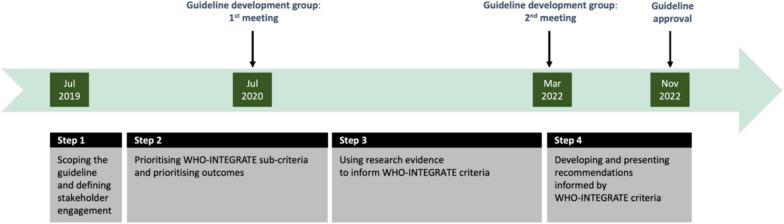
WHO parenting guidelines: development timeline

Figure 1 provides a chronological overview of the WHO parenting guideline development process. The decision in mid-2019 to develop guidelines was driven by the goal of preventing child maltreatment, aligning with the Sustainable Development Goals' target to end violence against children by 2030 [12] and the WHO’s 13th General Programme of Work's target to reduce such violence by 20% by the end of 2025 [13]. Parenting interventions are one of seven evidence-based strategies in the INSPIRE technical package for ending violence against children [14], recommended by WHO and other international agencies. Their relatively high degree of manualisation, their adaptability for different settings and the substantial evidence of their effectiveness made it an opportune time to create guidelines. This is to ensure that the efforts to deliver parenting interventions adhere to the highest evidence-based standards. In early-2020, the WHO secretariat selected the evidence synthesis team, focusing on its capability to conduct systematic reviews aligned with the WHO-INTEGRATE criteria. Additionally, two methodologists with expertise in GRADE and the WHO-INTEGRATE framework were recruited. Two virtual GDG meetings that took place in July 2020 and in March 2022 were the key forum for making guideline scope- and methods-related decisions, and for formulating guideline recommendations. The guideline was published in December 2022.

A WHO-internal planning proposal was submitted to the WHO Guidelines Review Committee in August 2020, with the revised proposal accepted in late October 2020. This described the scope, objectives and target audiences of the guidelines, specified the composition of the WHO steering group, the GDG and its two co-chairs, and the external review group, formulated initial guideline questions and outlined evidence synthesis methods to answer these questions. This planning proposal also described the use of the WHO-INTEGRATE framework throughout the guideline development process. Below, we provide a detailed description of each step, as actually implemented.

### Step 1: Scoping the guideline and defining stakeholder engagement

#### Processes and contributors

For the WHO parenting guidelines, this step was initiated by the WHO secretariat, mostly through development of the WHO-internal planning proposal. Within the guideline’s core team, multiple meetings were held to discuss the guideline’s scope, leading to refinements of the planning proposal. Decisions on the scope were eventually made by the GDG. Key processes included:

- **Choice of EtD framework:** The core team felt that a system-based whole-of-society approach, as reflected in the WHO-INTEGRATE framework, was suitable for the guideline, emphasising inter-sectoral parenting interventions with impacts beyond health.
- **Evidence mapping:** Given the vast literature on parenting interventions, especially numerous randomised controlled trials (RCTs), evidence mapping by the evidence synthesis team was chosen as a pivotal preparatory step to guide GDG discussions on the guideline scope, including identification of key gaps to inform further evidence synthesis (see Step 3 below).
- **Development of logic models:** The guideline methodologists, assisted by the WHO secretariat, created a system-based logic model that showcased relevant PICO and system elements (see online supplementary **Figure S1**). Additionally, a process-based logic model was developed to display short- and long-term outcomes for children and their parents, encompassing harsh and maltreating parenting, child behaviour and wellbeing, and social dimensions. Since existing models from the literature were not considered suitable, input was sought from selected parenting experts within the GDG.
- **Preliminary outcome listing:** The WHO secretariat developed an initial list of outcomes based on the process-based logic model, insights from the evidence map, and discussions among the core team.
- **GDG deliberation:** All preparatory findings were presented during the first GDG meeting where the GDG reviewed, deliberated, and finalised the guideline's scope, including the guideline questions.

#### Outputs and added value of WHO-INTEGRATE framework

The GDG for the WHO parenting guidelines featured diverse stakeholders, from scientists representing various disciplines to government officials, programme implementers, and civil society representatives across five WHO regions. Consensus emerged on the need for a complex systems and whole-of-society perspective, which would in part be realised through application of the WHO-INTEGRATE framework. Overall, it was agreed that the guideline would make five recommendations related to parenting interventions in distinct age groups of children (e.g. children vs. adolescents) and contexts (e.g. global vs. LMIC vs. humanitarian settings within LMICs). Accordingly, five questions were formulated to examine intervention effects on a broad range of outcomes in the specified age groups and contexts. In addition, the recommendations would also be informed by other WHO-INTEGRATE criteria/sub-criteria [2].

#### Challenges

Implementing this step requires a substantial time investment, both by the core team and by the GDG. A significant challenge was the limited time available for in-depth discussions within the GDG about system elements and broader topics (i.e., based on WHO-INTEGRATE criteria) that would require attention in the development of recommendations. The first GDG meeting focused largely on defining the PICO elements. Additionally, the logic models crafted to guide the process were not fully integrated but primarily served as tools for directing thought and defining scope. For example, when discussing guideline outcomes, GDG researchers and practitioners in parenting found the categorisation of outcomes as either short-term or long-term to be inappropriate due to most trials including outcomes that change in the short-to-medium term, and few including longer term outcomes that differ from these. Consequently, the use of a process-based logic model was discontinued.

### Step 2: Prioritising WHO-INTEGRATE sub-criteria and prioritising guideline outcomes

#### Processes and contributors

For the WHO parenting guidelines, the prioritisation of WHO-INTEGRATE sub-criteria by the GDG was conducted informally. In contrast, guideline outcomes were prioritised through a formal ranking method. Key processes included:

- **Discussion and prioritisation of WHO-INTEGRATE criteria and sub-criteria:** During the first GDG meeting, guideline methodologists introduced GDG members to the WHO-INTEGRATE framework, and its criteria and sub-criteria. The GDG considered all sub-criteria in a step-by-step manner.
- **Outcome ranking:** During the first GDG meeting, GDG members discussed the initial list of outcomes. After the meeting, they were engaged in an online survey to rank their importance. Following the GRADE methodology, outcomes were ranked on a scale from one to nine: unimportant (1–3), important (4–6), and critical (7–9).

#### Outputs and added value of the WHO-INTEGRATE framework

Table 1 presents the prioritised sub-criteria. Most were deemed important for the guideline, with only a few sub-criteria considered irrelevant; a minor change, combining two sub-criteria into one, was suggested by the GDG. Thinking through each of the WHO-INTEGRATE sub-criteria facilitated engagement with a complexity perspective, and considerations of the unintended consequences of an intervention beyond health. Six main categories of outcomes were eventually prioritised, all as critical, including child maltreatment, positive parenting skills and behaviour, harsh and negative parenting, child internalising problems (e.g. anxiety), child externalising problems (e.g. aggression, drug use), and parental mental health and stress.

#### Challenges

A primary challenge was insufficient time to comprehensively review all sub-criteria during a single meeting. With little prior experience with EtD frameworks, the GDG was somewhat overwhelmed by the number of WHO-INTEGRATE sub-criteria, leading to superficial discussions that often only yielded "yes/no" decisions regarding relevance and thus prioritising the majority of sub-criteria. The survey on outcome ranking prioritised 15 outcomes as “critical” or “important”. The core team, in consultation with the guideline co-chairs, had to make post-hoc adjustments (by way of regrouping outcomes) to limit the number of prioritised outcomes to six; these were subsequently approved by the GDG. Also, the GDG did not explicitly consider WHO-INTEGRATE criteria– beyond those directly related to health benefits and harms – in the ranking of outcomes. While the initial list of outcomes was informed by a complex systems perspective (mostly through the logic model), the ranking of outcomes did not explicitly take this into account.

### Step 3: Using research evidence to inform WHO-INTEGRATE criteria

#### Processes and contributors

Due to time constraints, in the WHO parenting guidelines, the GDG was not consulted on how to operationalise WHO-INTEGRATE sub-criteria in the guideline or determine the type of evidence synthesis needed to address them. Methodological decisions were made by the core team, with input from the guideline chairs, informing subsequent work by the evidence synthesis team. Key processes involved:

- **Translation of prioritised sub-criteria into research questions:** After the first GDG meeting, the core team worked on framing the prioritised sub-criteria as research questions for subsequent evidence synthesis. These questions extended beyond the effectiveness of parenting interventions to broader considerations, such as socio-cultural acceptability and the affordability and equity effects of such interventions across different contexts.
- **Deliberations on evidence synthesis approaches**: The core team evaluated the most suitable evidence synthesis approaches and/or more pragmatic rapid approaches. This phase involved several rounds of iterative discussions.
- **Commissioning and conduct of evidence syntheses:** Once the research questions and synthesis approaches were finalised, the reviews were formally commissioned following a request for proposals issued by the WHO secretariat.

#### Outputs and added value of WHO-INTEGRATE framework

**Fig. 2:**
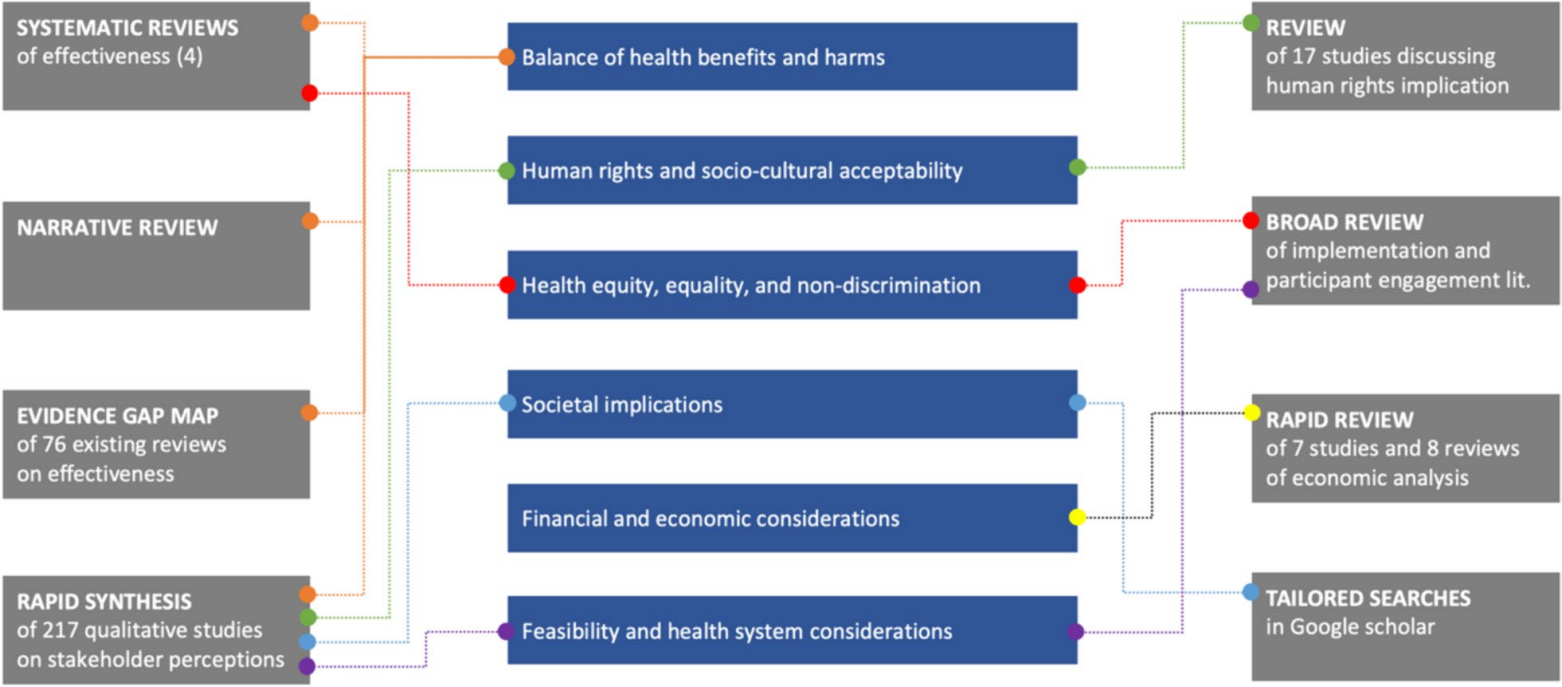
WHO parenting guidelines: Evidence synthesis products informing various WHO-INTEGRATE criteria for a given recommendation

Figure 2 illustrates the evidence synthesis products prepared to inform the WHO-INTEGRATE criteria [7, 8], enabling the integration of a complexity perspective with an evidence-based approach. Together, these represent a “system map of the research field”; individually, they are useful stand-alone resources. They comprised systematic reviews to assess the effectiveness of parenting interventions, and several more tailored approaches, such as an evidence map of existing systematic reviews on parenting interventions (see Step 1) and rapid mixed-method evidence syntheses to quickly gather insights on specific issues. For example, to assess potential harms, rapid synthesis of stakeholder perspectives was combined with data from the effectiveness reviews. Similarly, to assess equity issues, a rapid review of existing demographic within-trial moderator analyses was combined with planned between-trial meta-analysis of moderators in the effectiveness reviews, and with extracted data on programme coverage of disadvantaged groups. Furthermore, to address human rights implications and economic analyses, targeted literature searches were conducted.

#### Challenges

We had to decide on manageable strategies to synthesise the extensive body of literature on parenting interventions, which then comprised around 450 RCTs across HICs (> 300) and LMICs (> 150). The decision to prioritise nearly all WHO-INTEGRATE sub-criteria led to a substantial workload for the evidence synthesis team, with the various reviews retrieving over 200 qualitative studies, and many additional studies and reviews pertaining to implementation, cost-effectiveness and equity questions. Fortunately, we had the support of an experienced review team, well-versed in the subject matter and methodologically versatile, who leveraged their previous work, and the data from the effectiveness reviews. Additionally, standard systematic review methodology was not practicable for all sub-criteria, leading to prolonged discussions within the core team to determine the most appropriate approaches for non-effectiveness questions. Approaches beyond evidence synthesis were only explored to a limited extent given the large body of existing literature. Whereas targeted surveys among policymakers and implementers across different countries could have offered valuable in-depth insights, time constraints made it unfeasible to conduct such surveys. Another specific hurdle was the need to consolidate multiple questions, each on different sub-criteria, into coherent "evidence products”. Ultimately, these products were cross-applied to inform various WHO-INTEGRATE criteria (see Fig. 2). Often, evidence pertaining to non-effectiveness questions was not critically appraised due to time constraints and the absence of established evidence rating tools.

### Step 4: Developing and presenting recommendations informed by WHO-INTEGRATE criteria

#### Processes and contributors

In this step, the core team engaged in a collaborative and iterative process to prepare for the 2nd GDG meeting, where a set of draft recommendations and the evidence supporting these were critically debated. Main processes involved:

- **Preparation of preliminary EtD tables**: The evidence review team developed preliminary EtD tables for each guideline question. This involved identifying and integrating evidence from multiple synthesis products to make initial judgments for each sub-criterion (see Fig. 2). Areas lacking evidence or containing controversial findings were identified for in-depth GDG deliberation. The development of these EtD tables was iterative, incorporating several rounds of feedback from the guideline methodologists.
- **Drafting recommendations**: Utilising the preliminary EtD tables, the WHO secretariat drafted guideline recommendations, detailing their rationale and implementation considerations. These drafts were then discussed and revised by the core team.
- **GDG deliberation**: Prior to the 2nd GDG meeting, all GDG members were provided with the preliminary EtD tables. During the meeting, findings from the effectiveness review and the proposed judgements for the WHO-INTEGRATE sub-criteria, along with the evidence supporting them, were presented. The GDG critically evaluated the evidence, deliberated the recommendations and debated the strength of the recommendations. Formal voting was not used.
- **Finalisation of the EtD tables and recommendations:** After the second GDG meeting, the core team addressed the GDG’s comments, advancing both the EtD tables and the recommendations. The revised recommendations, including their specific wording, rationale, and implementation considerations, were circulated back to the GDG for written review and feedback.

#### Outputs and added value of WHO-INTEGRATE framework

**Fig. 3:**
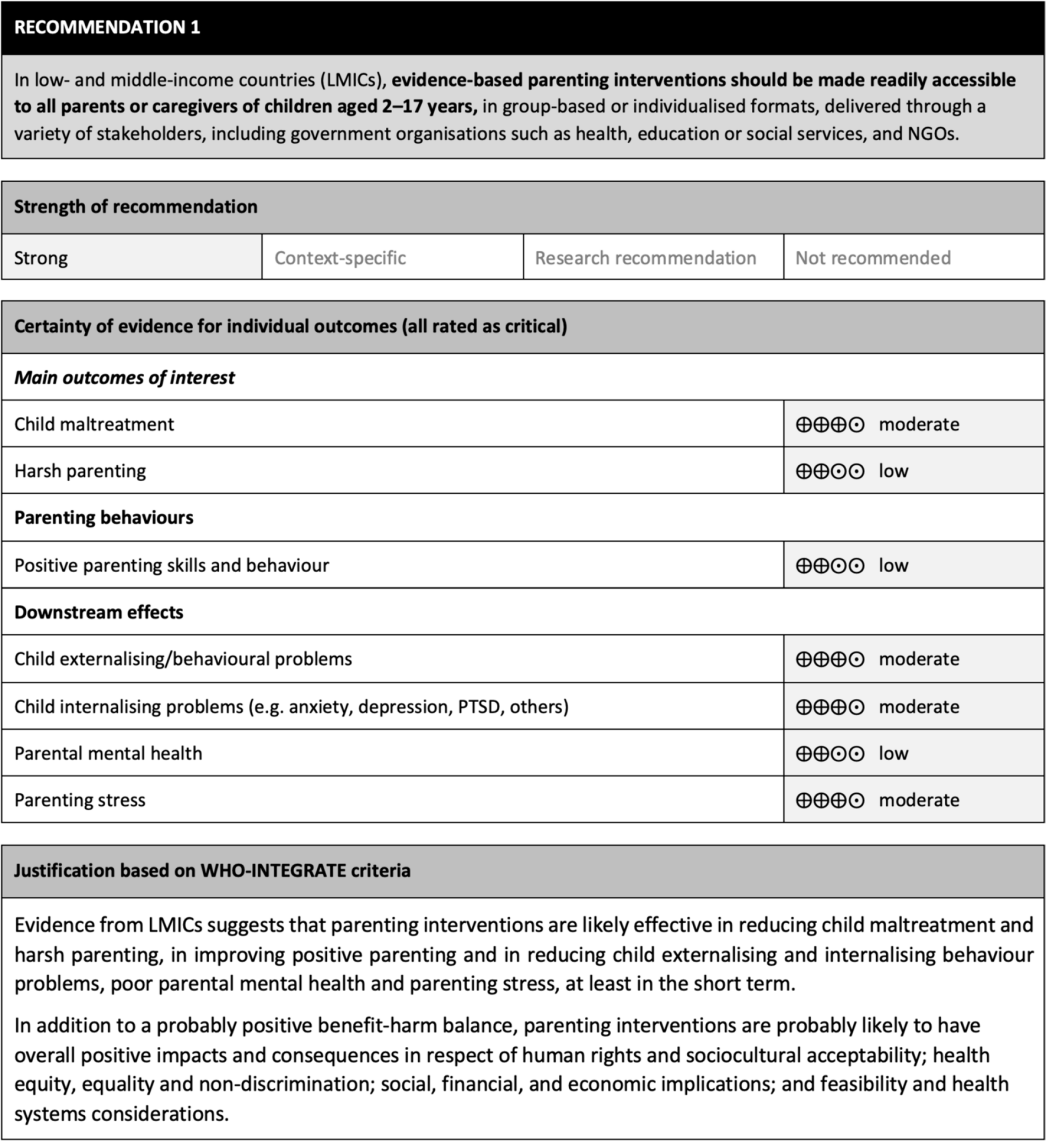
WHO parenting guidelines: Example recommendation

Five EtD tables, accompanying the five recommendations, were developed for the WHO parenting guidelines. Figure 3 illustrates the format used to present the recommendations in the guideline document [6]. It includes the recommendation, its strength, and an underpinning rationale, based on consideration of the WHO-INTEGRATE criteria and thus rooted in a whole-of-society approach. This comprises the certainty of evidence ratings for critical outcomes as per the GRADE approach and a summary paragraph that encapsulates the judgements made regarding the WHO-INTEGRATE criteria—these judgements are informed both by the gathered evidence and the GDG deliberations.

#### Challenges

A primary challenge in developing the EtD tables for the WHO parenting guidelines was condensing a substantial volume of evidence, sourced from a variety of evidence synthesis products, into a concise and comprehensible format. Despite the overall large volume of evidence, the insights related to parenting interventions in some age groups (e.g. adolescents) or settings (e.g. humanitarian settings) was limited; where this was the case the core team relied on indirect evidence obtained for different populations and settings. This approach appears justifiable in view of good transportability across populations of quantitative findings pertaining to the effectiveness of interventions, and the saturation observed for the qualitative findings pertaining to, for example, the acceptability and harms of interventions. Development of EtD tables necessitated multiple iterations, involving extensive review and feedback within the core team. While this method is efficient and commonly employed in WHO guideline development, it limits the opportunity for a truly collaborative co-development process, where the GDG plays a more active role in shaping the EtD tables and making judgments on WHO-INTEGRATE criteria. When determining the strength of a recommendation, the tendency to focus on the health benefits and harms – thereby underemphasising other considerations – presented a further challenge. For instance, many recommendations had GRADE certainty ratings for critical outcomes ranging from “moderate” to “low” (Fig. 3). Despite judgments in favour of a recommendation on several other WHO-INTEGRATE criteria, there were reservations about issuing “strong” recommendations.

## Discussion

### Value added by using the WHO-INTEGRATE framework in the WHO parenting guidelines

This paper has employed a multifaceted analysis, integrating guidance on the intended application of the WHO-INTEGRATE framework, introspective sessions in developing WHO parenting guidelines, and knowledge gained from a methodological workshop. This approach provides a rich description of the application of the framework’s theoretical constructs [10] in a real-world guideline development process. It highlights the value added and challenges encountered, offering a unique perspective and nuanced insights into guideline development in the fields of public health, health systems and health policy. However, this approach is not without limitations. Intrinsic biases may arise from introspective sessions, as reflections and interpretations are inherently subjective. Moreover, the workshop's methods-focused participant group might have constrained the diversity of perspectives, especially from those with less technical backgrounds. Despite these limitations, the paper contributes valuable lessons to the field.

Application of the WHO-INTEGRATE framework in the WHO parenting guidelines was crucial in integrating quantitative assessments of the effectiveness of parenting interventions with qualitative and, to a lesser extent, quantitative insights on their implementation and broader implications. This blend addresses the 'what' and the 'how' of parenting interventions, fostering a whole-of-society perspective that encompasses both intended and possible unintended health and non-health outcomes. The evidence reviews, informed by the WHO-INTEGRATE framework and summarised in the EtD tables, were instrumental during GDG discussions. They clarified uncertainties and informed key sections of the guideline, including justifications, subgroup recommendations, context and system considerations, implementation aspects, and research priorities. The evidence reviews synthesised a wide array of evidence, creating a 'system map' of the entire parenting research field and facilitated a more comprehensive view of “evidence”, extending beyond health benefits and harms. The initial evidence gap map helped identify critical areas for future research, such as cost implications of parenting interventions. Incorporating these multifaceted considerations into each recommendation, and including a chapter summarising the common WHO-INTEGRATE framework elements across all five recommendations, the WHO parenting guidelines answer whether parenting interventions are effective and delve into the complexities of their implementation across varied contexts.

### Challenges encountered while using the WHO-INTEGRATE framework in the WHO parenting guidelines

In applying the WHO-INTEGRATE framework to the development of WHO parenting guidelines, several challenges were encountered. A primary difficulty was the substantial time investment required by all involved, particularly evident in the scoping phase and in the comprehensive consideration of the numerous WHO-INTEGRATE sub-criteria. The GDG did not have sufficient time for in-depth discussions, impacting their ability to thoroughly review the various system elements and choose the most relevant sub-criteria. Furthermore, the extensive body of literature on parenting interventions was difficult to synthesise, especially given the broad scope of the prioritised sub-criteria. Operationalising these sub-criteria in the guideline and determining the appropriate type of evidence synthesis also posed difficulties. Additionally, the process of integrating a diverse array of evidence into coherent, concise, and comprehensible formats for guideline recommendations was a complex task without straightforward guidance being available. This required the core team’s expertise, adaptability, and multiple iterations. The need to condense varied evidence synthesis products and the reliance on indirect evidence for certain age groups or settings required meticulous judgment and consideration.

### Recommendations for the development of guidelines seeking to use the WHO-INTEGRATE framework

The challenges encountered and experiences in developing the WHO parenting guidelines have yielded valuable lessons and specific recommendations for GDGs/guideline panels and guideline methodologists wishing to use the WHO-INTEGRATE framework. These insights, applicable to the guideline development process as a whole or across specific steps of the process, are summarised below and detailed in Table 3.

**Table 3:** Application of the WHO-INTEGRATE framework: lessons learnt from the WHO parenting guidelines, overall and for steps 1–4

Lessons learnt	Planning and resources	Processes, tools and methods
Overall	**EXPERTISE:** Make methodological and interdisciplinary expertise a core criterion when selecting and working with the evidence synthesis team(s) and methodologist(s) **WORKLOAD:** Plan for a high workload for the core team, notably the WHO secretariat, evidence synthesis team(s) and methodologist(s), taking into account that all steps usually involve some degree of iteration **GDG/GUIDELINE PANEL COMPOSITION:** Make sure that the GDG/guideline panel composition appropriately reflects all aspects relevant to the development of the guideline, notably with regards to WHO-INTEGRATE criteria and sectors affected beyond health **GDG/GUIDELINE PANEL TIME COMMITMENT:** Consider a high GDG/guideline panel time commitment prior to, during and after meetings when planning for and structuring meetings	**MINDSET:** Encourage a broad mind-set beyond a consideration of health benefits and harms among all involved with guideline development and throughout the guideline development process **TOOLS:** Use the various tools and approaches to facilitate a complexity perspective—e.g. WHO-INTEGRATE sub-criteria, logic modelling and GDG/guideline panel deliberation—in a reasonable, pragmatic manner to maintain efficiency **METHODS:** Choose appropriate methods to back WHO criteria and sub-criteria with evidence, evaluating the possibility of different evidence synthesis and pragmatic approaches and leveraging existing evidence wherever possible **PROCESSES:** Encourage more collaborative processes with direct involvement of GDG/guideline panel members to develop recommendations backed by WHO-INTEGRATE criteria; balance engagement from an early stage with efficiency considerations **PRESENTATION:** Present the evidence and deliberations backing WHO-INTEGRATE criteria in an accessible and transparent manner, tailoring the balance between comprehensiveness and conciseness to the needs of different user groups, such as GDG/guideline panel members vs. guideline users
Step 1	**EXPERTISE:** Recruit methodologist(s) with WHO-INTEGRATE framework expertise; involve the evidence synthesis team(s) with expertise in various evidence synthesis approaches and the content area **WORKLOAD:** Implement a phased approach to scoping. Allocate sufficient time and resources for this iterative process, including for commissioning preparatory evidence review projects (e.g. scoping review or evidence gap map) **GDG/GUIDELINE PANEL COMPOSITION:** Recruit GDG/guideline panel members who bring expertise with regards to various WHO-INTEGRATE criteria, such as economic implications, legal considerations, and cultural competencies **GDG/GUIDELINE PANEL TIME COMMITMENT:** Share resources on the WHO-INTEGRATE framework for GDG/guideline panel members in preparation for the 1st meeting; and/or allocate a session dedicated to the framework during the 1st meeting	**MINDSET:** Incorporate sessions for GDG/guideline panel members prior to/during the 1st meeting that emphasise the importance of considering a wide array of factors, health equity, cultural relevance, feasibility, and economic implications, alongside health benefits and harms **TOOLS:** Select and utilise tools that directly contribute to defining the scope and objectives in a given guideline. For instance, use logic models to map out the system elements and broad impacts when deemed helpful
Step 2	**WORKLOAD:** Allocate dedicated time and resources for preparing for the 1st GDG/guideline panel meeting; involve the evidence synthesis team(s) and methodologist(s) in these preparations **GDG/GUIDELINE PANEL TIME COMMITMENT:** Allocate sufficient time for the WHO-INTEGRATE criteria discussions during the 1st GDG/guideline panel meeting	**MINDSET:** During the 1st meeting, encourage GDG/guideline panel members to consider broader impacts like societal benefits, feasibility, and acceptability of interventions. Facilitate a balanced consideration of various aspects in the prioritisation process **METHODS:** Select an approach of prioritising sub-criteria that suits GDG/guideline panel needs and preferences, e.g. informal GDG/guideline panel discussions vs. more structured scoring approaches **PROCESS:** Consult GDG/guideline panel members during the 1st meeting on the most relevant and feasible approaches for evidence synthesis
Step 3	**WORKLOAD:** Plan for additional evidence synthesis projects (e.g. qualitative evidence synthesis, review of ethics literature) and pragmatic approaches beyond effectiveness reviews	**MINDSET:** Diversify the types of evidence to be synthesised. Encourage the evidence synthesis teams to look for and incorporate findings from qualitative studies, policy analyses, and socio-economic research **METHODS:** Prioritise systematic vs. rapid/targeted evidence synthesis approaches for various criteria considering the timeline of the guideline and feasibility; use/update existing reviews
Step 4	**WORKLOAD:** Plan for additional time for developing the EtD tables **GDG/GUIDELINE PANEL COMPOSITION:** Facilitate GDG/guideline panel discussions that give equal consideration to effectiveness and other prioritised WHO-INTEGRATE criteria/sub-criteria, each backed by the appropriate type of evidence or, where no evidence is available, expertise **GDG/GUIDELINE PANEL TIME COMMITMENT:** Allocate more dedicated time for the WHO-INTEGRATE criteria discussions during the final GDG/guideline panel meeting	**MINDSET:** In drafting recommendations, explicitly require the inclusion and discussion of criteria beyond health benefits and harms **METHODS:** In informing criteria in the EtD tables, draw on one or multiple evidence synthesis products, re-using insights and labelling these as “indirect evidence”, when appropriate. During the development of recommendations, focus GDG/guideline panel deliberations on criteria/judgements lacking evidence and report these transparently **PROCESSES:** Divide the GDG/guideline panel into smaller working groups and involve them in drafting the EtD tables and recommendations prior to the final GDG/guideline panel meeting; discuss the recommendations in the wider group and adapt them based on the feedback received **PRESENTATION:** Clearly structure the EtD tables, for example, by highlighting the sources of judgement for each criterion (e.g. evidence vs. GDG/guideline panel deliberation) and by including a brief summary of the synthesised evidence. Link these to more extensive evidence synthesis outputs

Step 1: Scoping the guideline and defining stakeholder engagement

Step 2: Prioritising WHO-INTEGRATE sub-criteria and prioritising outcomes

Step 3: Using research evidence to inform WHO-INTEGRATE criteria

Step 4: Developing and presenting recommendations informed by WHO-INTEGRATE criteria

**Step 1: Scoping the guideline and defining stakeholder engagement:** For this first step of guideline development, an emphasis on comprehensive scoping is essential. In our experience, an iterative approach to defining the scope and questions might be helpful, incorporating a range of expertise in the GDG/guideline panel. Such diversity should reflect relevant WHO-INTEGRATE criteria, ensuring a broad perspective from the outset. Furthermore, engaging stakeholders early in the process, including those directly impacted by the guidelines, is crucial for ensuring relevance and applicability.

**Step 2: Prioritising WHO-INTEGRATE sub-criteria and guideline outcomes:** In the second step, dedicated sessions for GDG/guideline panel discussions on WHO-INTEGRATE sub-criteria are recommended. It is important to prepare GDG/guideline panel members in advance, emphasising a broad mind-set that extends beyond health benefits and harms. This preparation can include educational resources or dedicated workshops on complex systems thinking in relation to public health, health system or health policy interventions and the WHO-INTEGRATE framework. The process of prioritising sub-criteria should be structured yet flexible, allowing for in-depth exploration and consensus-building.

**Step 3: Using research evidence to inform WHO-INTEGRATE criteria:** For the third step, streamlining evidence synthesis is advised. “Game-changing” sub-criteria, whether relating to effectiveness or broader questions, must be supported by rigorous evidence, and a focus on these critical criteria can also help manage the scope of evidence synthesis and ensure feasibility. Utilising existing systematic reviews can significantly reduce the time and effort required for new syntheses. Additionally, flexibility in choosing evidence synthesis or more pragmatic and thus more rapid approaches is critical, thereby keeping the timeliness and feasibility of the guideline development process in mind.

**Step 4: Developing and presenting recommendations informed by WHO-INTEGRATE criteria:** In the final step, developing strategies for efficiently distilling extensive evidence on various WHO-INTEGRATE criteria into concise and accessible formats is vital. This may include creating tailored summaries for different user groups—detailed versions for GDG/guideline panel members and concise summaries for broader guideline users. Facilitating collaborative co-development by actively involving GDG/guideline panel members in early-stage work on the EtD tables can enhance the quality and acceptance of the recommendations.

Across all steps, balancing the depth of WHO-INTEGRATE criteria discussions with efficiency and maintaining transparency in presenting evidence and deliberations is important. These recommendations, derived from the experience with applying the WHO-INTEGRATE framework in the WHO parenting guidelines and from the insights during a methodological workshop, aim to streamline the use of the WHO-INTEGRATE framework in the guideline development process, while ensuring a comprehensive approach. Such a comprehensive approach is important to pay tribute to the complexity of public health, health system and health policy interventions and their broader societal impacts.

### Supplementary Information

Supplementary Material 1.

## Data Availability

All data generated or analysed during this study are included in this published article and its supplementary information file.
