# Supplementary material for: Applying the WHO-INTEGRATE evidence-to-decision framework in the development of WHO guidelines on parenting interventions: step-by-step process and lessons learnt
Source: Health Res Policy Syst. 2024 Jul 5;22:79. doi: 10.1186/s12961-024-01165-z (PMC11227174; doi:10.1186/s12961-024-01165-z)

# Supplementary materials

**Figure S1:** System-based logic model used to scope WHO parenting guidelines
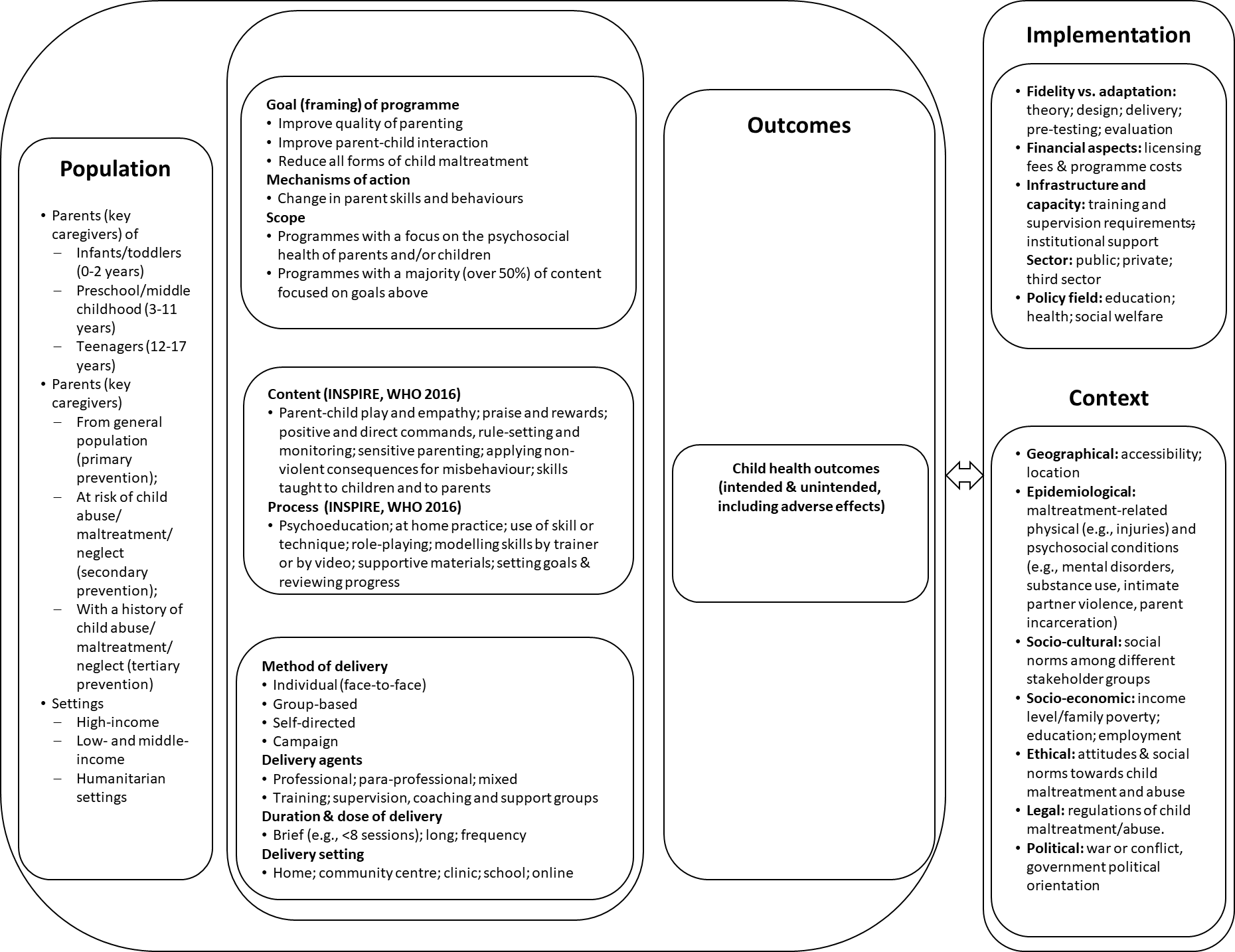

Supplement: Supplementary file 1 — Supplementary Material 1. [file 12961_2024_1165_MOESM1_ESM.docx]
